# New Perspectives on the Evolutionary History of Vitellogenin Gene Family in Vertebrates

**DOI:** 10.1093/gbe/evy206

**Published:** 2018-09-18

**Authors:** Maria Assunta Biscotti, Marco Barucca, Federica Carducci, Adriana Canapa

**Affiliations:** Dipartimento di Scienze della Vita e dell’Ambiente, Università Politecnica delle Marche, Ancona, Italy

**Keywords:** vitellogenin, vertebrates, gene family evolution

## Abstract

Vitellogenin (Vtg) is a glycolipophosphoprotein produced by oviparous and ovoviviparous species and is the precursor protein of the yolk, an essential nutrient reserve for embryonic development and early larval stages. Vtg is encoded by a family of paralog genes whose number varies in the different vertebrate lineages. Its evolution has been the subject of considerable analyses but it remains still unclear. In this work, microsyntenic and phylogenetic analyses were performed in order to increase our knowledge on the evolutionary history of this gene family in vertebrates. Our results support the hypothesis that the vitellogenin gene family is expanded from two genes both present at the beginning of vertebrate radiation through multiple independent duplication events occurred in the diverse lineages.

## Introduction

In vertebrates vitellogenin (Vtg) is a high molecular weight (300–640 kDa) glycolipophosphoprotein typically present in female but also in minor amounts in males (Canapa et al. 2007; Barucca et al. 2010; Canapa et al. 2012; Verderame and Scudiero, 2017), produced by oviparous and ovoviviparous species during the reproductive process as it is the precursor protein of the yolk, an essential nutrient reserve for embryonic development and early larval stages.

In general, the vitellogenin amino acidic sequences are made up of a signal polypeptide, a heavy chain lipovitellin (LvH) including four subdomains (N sheet, α-helix, C sheet, and A sheet), a phosvitin (Pv), a light chain lipovitellin (LvL), and a von Willebrand factor type D domain (Vwfd) containing a beta component (β′) and a C-terminal coding region (CT). The vtg C of teleost lack of Pv domain (Canapa et al. 2012).

The synthesis of vtg protein occurs in hepatocytes (heterosynthesis) and the produced protein is phosphorylated, glycosylated, and lipid groups are added before being released into the bloodstream that transports it to the ovary where it passes between ovarian follicular cells and is incorporated into the ovaries by receptor-mediated endocytosis (Patiño and Sullivan, 2002).

In mature oocytes vitellogenin is disrupted in multiple proteins: The lipovitellin, a lipoprotein that contains phosphorus, lipids, carbohydrates, and in fish also calcium and iron, the Pv, highly phosphorylated, the β′ component, and the C-terminal peptide (Finn and Kristoffersen, 2007; Sun and Zhang, 2015; Hara et al. 2016).

Vitellogenin is also produced in invertebrate chordates (Akasaka et al. 2013) and in invertebrates such as molluscs (Agnese et al. 2013; Chen et al. 2018) and arthropods (Hannas et al. 2011; Wu et al. 2018) in which this protein is synthetized in oocytes (autosynthesis) or in different districts (heterosynthesis).

Experimental evidence has showed that vitellogenin is encoded by a family of paralog genes whose number varies in the different vertebrate lineages. In the jawless silver lamprey *Ichthyomyzon unicuspis* a single gene is present and a single sequence has been identified in the catshark *Scyliorhinus torazame* (Yamane et al. 2013). Instead, three sequences of vitellogenin are present in nonteleost fish, the spotted gar *Lepisosteus oculatus* and the bichir *Acipenser schrenckii*. Among teleosts, salmonids have three paralog genes coding for *vtgAsa1*, *vtgAsb*, and *vtgC* (Buisine et al. 2002; Finn and Kristoffersen, 2007; Andersen et al. 2017). Cyprinids and anguillids have several homologous genes coding for vitellogenins attributable to v*tgAo* and *vtgAe*, respectively (Finn and Kristoffersen, 2007). Acanthomorpha present at least three different genes of vitellogenin: *vtgAa*, *vtgAb*, and *vtgC* (Matsubara et al. 2003; Hiramatsu et al. 2006; Finn and Kristoffersen, 2007). The latter, unlike those hitherto cited, is an incomplete form of vitellogenin as it lacks the Pv domain and has a truncated C-terminal end, and thus existing only as a complex LvH–LvL (Hiramatsu et al. 2005, 2006; Finn and Kristoffersen, 2007). To date, up to three coding genes for vitellogenin have been identified in the sarcopterygian lineage (Finn and Kristoffersen, 2007; Babin 2008; Brawand et al. 2008; Canapa et al. 2012).

Several studies have investigated the evolutionary history of the vitellogenin gene family. In 2007, Finn and Kristoffersen hypothesized that the presence of multiple copies of vitellogenin in the genome was due to whole genome duplication (WGD) events. Through the WGD, the genome undergoes a duplication of each component, the genes thus present in two copies can acquire new functions. In vertebrates four events of genomic duplication occurred: 1R and 2R at the stem of vertebrates (Smith et al. 2013), the teleost-specific WGD (Ts3R) at the base of teleosts (Jaillon et al. 2004; Kasahara et al. 2007; Nakatani et al. 2007), and the salmonid-specific WGD (Ss4R) in the common ancestor of salmonids (Near et al. 2012; Macqueen and Johnston 2014). Therefore early-branching fish and tetrapods would have had four genes of vitellogenin, while in teleosts eight genes were expected and 16 *vtg* genes in salmonids. Losses of genes following WGDs and specific polyploid phenomena in certain taxa would have given different results than those expected (Finn and Kristoffersen, 2007).

In 2008, based on phylogenetic and syntenic studies, Babin advanced the hypothesis of the existence of an ancestral gene cluster composed of three vitellogenin genes, originated before the separation of teleosts and tetrapods. Starting from the chromosomal localization of the three available *Gallus gallus* vitellogenin (*vtgI*, *vtgII*, *vtgIII*) sequences, Babin observed that in some teleost genomes three *vtg loci* homologues for synteny were present. The chromosomal distance between the *locus vtgI* (putative orthologous of the teleost *vtgC* gene) and the *vtgII*/*vtgIII loci* suggested that these genes originated from the duplication of a single ancestral gene. Finn et al. (2009) and Kristoffersen et al. (2009) recognized the validity of the vitellogenin gene cluster proposed by Babin (2008) and reformulated their previous model.

Another study on tetrapods, focused on the loss of vitellogenin genes in placental mammals (which occurred parallel to the development of new reproduction strategies and embryonic growth), has suggested a hypothesis on the evolution of this family based on both the presence of an ancestral cluster and gene duplication events (Brawand et al. 2008). According to this hypothesis before the reptile/amphibians split, the genes would have been only two, *vitI* (*vtgI* in the nomenclature suggested by Babin) and *vitanc* (*vtg* ancestral); the latter would have originated *vtgII* and *vtgIII* through duplication events in the various taxonomic groups.

In 2012 Canapa and colleagues, after the isolation and analysis of the mRNA of *Latimeria menadoensis*, taxon considered to be external to Rhipidistians, identified three different vitellogenins. The phylogenetic analysis performed comparing *Latimeria* sequences with those of the major oviparous/ovoviviparous vertebrate groups showed that one of the identified sequences was orthologous to those of tetrapods (*vtgI*) and constituted a gene phylogenetically separated compared with the other two.

Moreover since vitellogenin gene family belongs to the large lipid transfer protein (LLTP) superfamily, it has been suggested a correlation with the major yolk protein toposome even if the question is still open (Prowse and Byrne, 2012; Dev and Robinson, 2014; Castellano et al. 2018).

In this work, microsyntenic and phylogenetic analyses were performed in order to increase our knowledge on the evolutionary history of the vitellogenin gene family in vertebrates. Here, four new sequences were obtained from the lungfish *Protopterus annectens*, the closest species to the tetrapod ancestor (Amemiya et al. 2013; Biscotti et al. 2016). The results obtained seem to support the hypothesis that the vitellogenin gene family is expanded from two genes both present at the time of the gnathostome radiation. Multiple independent duplication events occurred in the diverse lineages.

## Materials and Methods

### Sequence Collection

Fifty-five *vtg* sequences belonging to 17 vertebrate species were collected from NCBI (https://www.ncbi.nlm.nih.gov/) or ENSEMBL (http://www.ensembl.org/index.html) databases. Four sequences of *Xenopus laevis* were collected from XENBASE (http://www.xenbase.org/entry/), three sequences of the Atlantic salmon *Salmo salar* were collected from Andersen et al. (2017), and the sequence of the lamprey *Petromyzon marinus* from the UCSC Genome Browser (http://genome.cse.ucsc.edu/). For *G.gallus*, *Pelodiscus sinensis*, *Latimeriachalumnae*, and *Oryzias latipes* the exon–intron arrangement was retrieved from ENSEMBL and NCBI. A complete sequence and six partial transcripts were retrieved from the *P. annectens* transcriptome (Biscotti et al. 2016). These sequences were completed by PCR, 3′-RACE and 5′-RACE (supplementary fig. S1, Supplementary Material online). The latter was carried out using the 5′-RACE System for Rapid Amplification of cDNA Ends kit, version 2.0 (Invitrogen, Carlsbad, CA) according to the manufacturer’s instructions. The sequence of the primers used in PCR and in RACE techniques are listed in supplementary table S1, Supplementary Material online. PCRs were performed in a thermal cycler using Platinum Taq DNA Polymerase (Invitrogen, Carlsbad, CA). The PCR products obtained from 5′ and 3′ RACE were cloned in pGEM-T Easy Vector (Promega, Madison, WI). The PCR products and the obtained clones were sequenced.

### Microsyntenic and Phylogenetic Analyses

The microsyntenic arrangement of the *vtg* genes was obtained from ENSEMBL for *Homo sapiens*, *G.gallus*, *P.sinensis*, *L.chalumnae*, *Callorhinchus milii*, *O.latipes*, and *Gasterosteus aculeatus*, from XENBASE for *X. laevis*, and from Salmobase (https://salmobase.org/) for *S. salar*. An accurate BLAST analysis was conducted on the genome of interest organisms in order to identify all the *vtg* genes present as well as those already annotated. Moreover, the synteny was checked through Genomicus (http://www.genomicus.biologie.ens.fr/genomicus-84.01/cgi-bin/search.pl).

The alignment was performed with ClustalW (http://www.genome.jp/tools-bin/clustalw) using default parameters. The phylogenetic analysis was carried out with MrBayes-3.2 (Huelsenbeck et al. 2001). On the basis of the results of microsyntenic analysis the sequences belonging to the *locus* vtgI/C were constrained to be monophyletic. The Jones aa model (Jones et al. 1992) was identified by the MrBayes program with a posterior probability of 1.00. The Vtg sequence of *I.unicuspis* was used as the outgroup; 6,000,000 generations were run and sampling was conducted every 100 generations. Stationarity was defined as the condition where the standard deviation of split frequencies reached 0.0004. The first 15,000 trees were discarded as the burn-in. The accession numbers of the used sequences are listed in supplementary table S2, Supplementary Material online.

## Results and Discussion

**Fig. 1.**
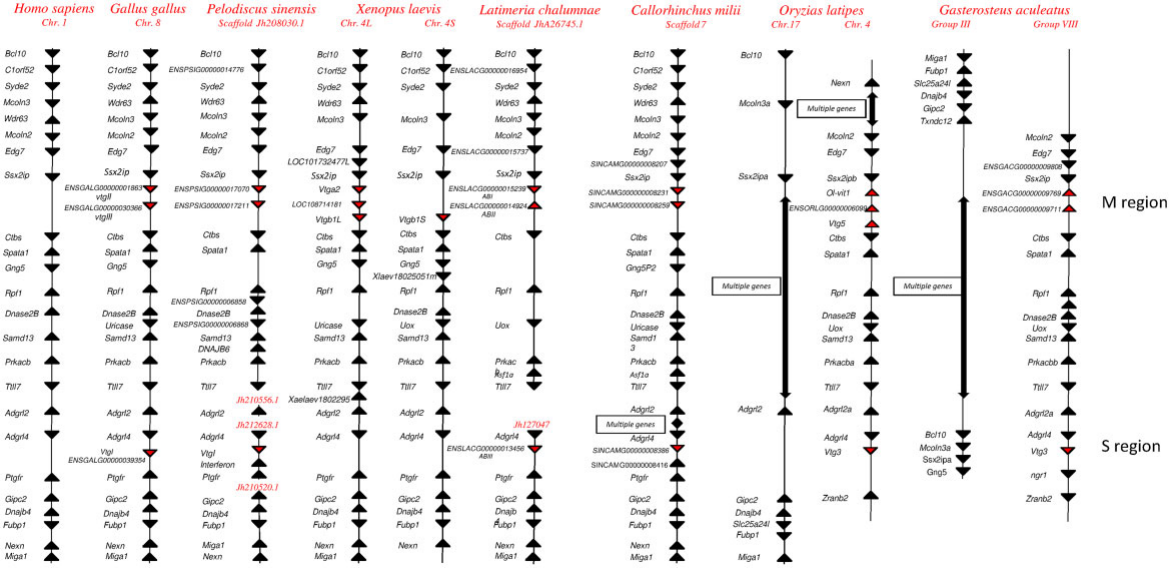
—Synteny conservation of *vtg* genes. Lines underneath genes indicate syntenic arrangement and arrowheads indicate gene direction. Syntenic maps are reported for *Homo sapiens*, *Gallus gallus*, *Pelodiscus sinensis*, *Xenopus laevis*, *Latimeria chalumnae*, *Callorhinchus milii*, *Oryzias latipes*, and *Gasterosteus aculeatus*. The gene distances are not in scale.

The microsynteny of chromosome regions harboring the *vtg* genes was investigated in the main vertebrate species (fig. 1). The analysis evidenced that the *vtg* genes are localized into two chromosomal regions, named here M region (multiple *vtg* genes) and S region (single *vtg* gene). In the M region up to three genes showing a tandem arrangement are present and probably derived from duplication events. The S region harbors the *vtgC* gene of teleosts and the *vtgI* gene of tetrapods. In human *vtg* genes are absent in both regions as in other therian mammals. In the genome of the tetraploid *X. laevis* the microsyntenic analysis allowed to identify three genes in the M region on the 4L chromosome and one on the 4S chromosome while in the S region *vtg* genes are absent on both chromosomes. In teleosts, the microsynteny of genes flanking the M and S regions showed a derived gene organization compared with cartilaginous fish and sarcopterygians. Indeed these genes are localized into two chromosomes probably due to the duplication event occurred in teleost genomes (TSGD) that associated with further rearrangements (Kasahara et al. 2007; Nakatani et al. 2007) have led to the preservation of the *vtg* genes in only one of the two chromosomes. In *S. salar* the *vtg* genes are present in two chromosomes and the microsyntenic arrangement results more complex since salmonids have undergone a further WGD (Ss4R) (supplementary fig. S2, Supplementary Material online).

**Fig. 2.**
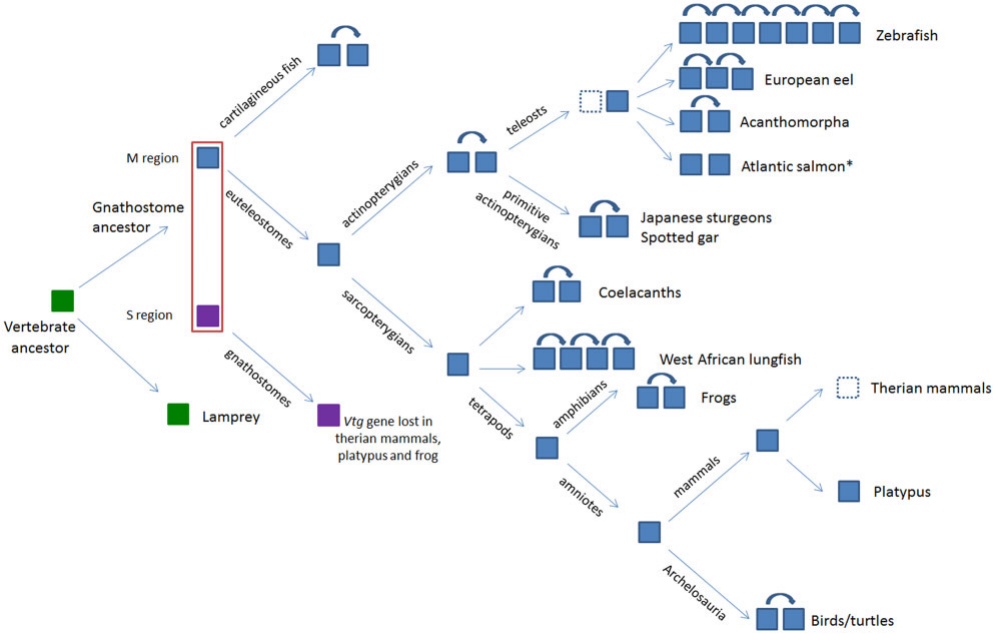
—Schematic representation of the hypothesis proposed for the evolution of *vitellogenin* in vertebrates. Curved arrows above filled squares indicate a tandem duplication event. Dashed square indicates gene loss. * The *vtg* two genes of Atlantic salmon are not due to tandem duplication event but to the salmonid-specific WGD.

Coelacanth and elephant shark, two organisms considered living fossils given the maintenance of ancestral features, share the same microsyntenic arrangement as also evidenced for other gene families in our previous reports ( Biscotti et al. 2017, 2018 ). The shared microsynteny between coelacanth and elephant shark suggests that the common ancestor of gnathostomes already had this gene organization. In the jawless lamprey a unique *vtg* gene has been identified suggesting that the duplication of the first *vtg* gene occurred in the ancestor of gnathostomes, before the separation between Osteichthyes and Chondrichthyes (fig. 2).

**Fig. 3.**
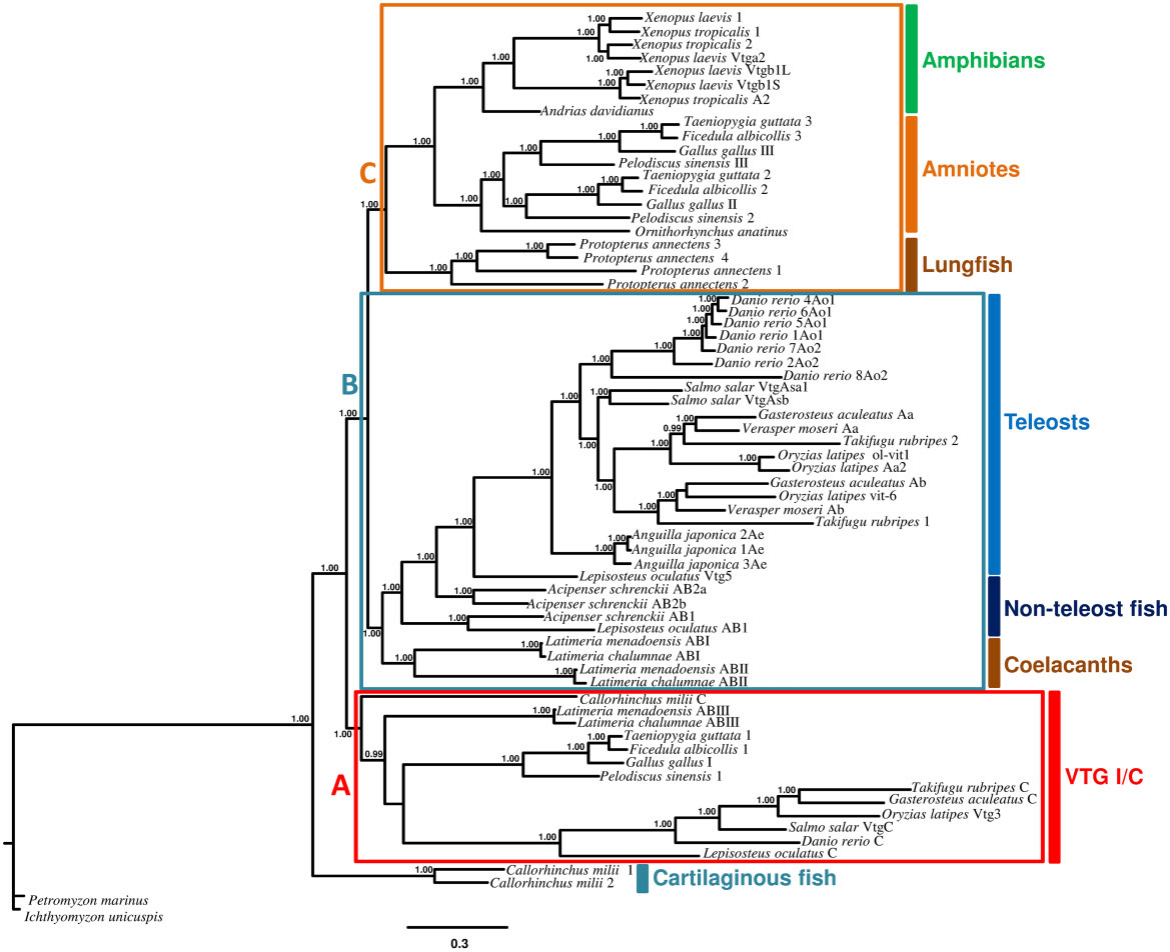
—Phylogenetic analysis of vitellogenin sequences of vertebrates obtained with Bayesian Inference. Number beside nodes indicates posterior probability values (>0.95). The silver lamprey *Ichthyomyzon unicuspis* was used as the outgroup.

The phylogenetic analysis, based on 66 gnathostome sequences belonging to 21 species and two agnathe sequences belonging to two species, shows two sequences of *C. milii* in the external position and the other sequences grouped into three clades: The A clade including VtgI of tetrapods, one sequence of elephant shark, one sequence for each *Latimeria* species, one sequence of spotted gar, and VtgC sequences of teleosts; the B clade including sequences belonging to coelacanth (in external position), spotted gar, sturgeons, and teleosts; the C clade including the Vtgs of lungfish and the VtgII and III of tetrapods. The A clade is located external to the other two clades (fig. 3).

In the C clade the duplication events that have led to multiple *vtg* genes in the M region occurred independently. It can be hypothesized as also suggested by Brawand et al. (2008) that at the time of separation of amphibians and amniotes in the M region there was a single gene that subsequently underwent lineage specific duplications. The external position of the platypus Vtg compared with the two subgroups constituted by VtgII and VtgIII of turtle and birds (Archelosauria Crawford et al. 2015) suggests that the duplication in Archelosauria occurred after the separation of the evolutionary lineage leading to mammals.

From the phylogenetic analysis it is also clear that the *vtg* genes present in the two species of *Xenopus* are orthologous and consequently the common ancestor already had this gene arrangement. Moreover, in *X. laevis* the proximity of Vtgb1L to Vtgb1S evidences that their orthology is due to the genome duplication event that affected this amphibian. Consequently, the absence of the other two genes in the M region on the 4S chromosome in *X. laevis* is due to secondary losses. This is in agreement with the recent analysis of the two subgenomes of the African clawed frog that showed how the S subgenome experienced more gene loss, deletion, rearrangement (Session et al. 2016). The absence of *vtgI* gene in the S region is also probably due to secondary loss.

The lungfish sequences form a species specific clade indicating that independent duplications occurred in the M region in this species.

Inside the B clade there are two subgroups: One constitutes by sequences of *Latimeria* and the other group by sequences of *Acipenser*, *Lepisosteus*, and those of teleosts. The position of coelacanth sequences in this clade is probably due to the primitive nature of its genome (Makapedua et al. 2011; Amemiya et al. 2013) that shares with the early-branching fish. Previously we have reported that even for cleavage site of protein (Canapa et al. 2012) the *Latimeria* sequences showed an intermediate condition between sturgeons and tetrapods. However, the tree evidences that the *vtg* sequences of *Latimeria* are the result of independent tandem duplications and this allows to anticipate the presence of a single gene in the M region already in the ancestor of sarcopterygians.

The division into two subgroups inside the B clade, one constitutes by one sequence of *Acipenser* and one of *Lepisosteus*, and the other subgroup by two sequences of *Acipenser*, one of *Lepisosteus*, and those of teleosts suggests that two genes were present in the M region of the Actinopterygian ancestor (fig. 2) and these genes remained in the spotted gar and in the sturgeon where in the latter one of the two genes underwent a species-specific duplication. In teleosts one of the two genes was lost while the other gene has been affected by gene duplication events. Indeed, the seven VtgAo of *D. rerio* as well as the three VtgAe of *Anguilla* constitute separate groups indicating species-specific duplications. In Acanthomorpha the duplication of *vtgA* gene that led to the current *vtgAa* and *vtgAb* genes occurred in the common ancestor of this taxon.

The comparison of the exon–intron arrangement of the *vtg* paralog genes in *G. gallus*, *P. sinensis*, *L. chalumnae*, and *O. latipes* evidences a more similar pattern between the genes located in the M *locus* compared with the *vtgC/I* gene of S *locus* (supplementary fig. S3, Supplementary Material online). This finding is in agreement with the microsyntenic and phylogenetic results and with the evolutionary hypothesis here proposed.

Overall the occurrence of several duplication events concerning this gene is probably related to the high versatility of the different proteins of Vtg in performing various functions. Indeed although Vtg, the precursor of yolk proteins, was traditionally regarded as the energy reserve for nourishment of the developing embryos, recently new roles have been reported. Hughes (2015) analyzing the Vtg sequences from 34 avian species suggests that the different amino acid compositions of Vtgs co-evolves with reproductive strategy in birds. The amino acid composition of Vtgs plays also a role in the buoyancy of eggs in Acanthomorpha teleosts. Indeed the proteolysis that affects the VtgA protein, typical of this taxon, seems to be related to the pelagic or benthic nature of the eggs (Finn et al. 2002; Kolarevic et al. 2008) since the produced free amino acids can influence the oocyte hydration. Several reports have also demonstrated that Vtg is an immune-relevant molecule involved in the host defense against the microbes including bacterium and virus (Zhang et al. 2011, 2015; Sun and Zhang, 2015). Indeed Vtg binds to lipopolysaccharide, lipoteichoic acid, peptidolycan, glucan, and virons (Shi et al. 2006; Liu et al. 2009) and acts as a bactericidal molecule capable of damaging bacterial cell walls (Sattar Khan et al. 2000).

## Conclusions

In conclusion the results of microsyntenic and phylogenetic analyses outline a picture (fig. 2) in agreement with the hypothesis that vitellogenin gene family expanded from two genes already present in the common ancestor of Gnathostomes. Moreover, the *vtg* gene has undergone several duplications during its evolutionary history that led to the formation of a gene family whose members are located into two chromosomal regions and one of them underwent tandem duplication events lineage specific.

## Author Contributions

M.A.B. and M.B. were involved in the phylogenetic analysis. M.A.B. and F.C. performed microsyntenic analyses. M.A.B. was involved in experimental procedures. M.B. and A.C. made contributions to the conception of the study and the experimental design. All authors interpreted the data, wrote the paper and have given final approval for the version to be published. This work was supported by a grant from Ministero della Ricerca e dell’Istruzione, Project number: 69, 2013. We thank the anonymous reviewers for their constructive comments, which helped us to improve the manuscript.

## Supplementary Material

Supplementary Data
