# Supplementary material for: New Perspectives on the Evolutionary History of Vitellogenin Gene Family in Vertebrates
Source: Genome Biol Evol. 2018 Sep 18;10(10):2709–15. doi: 10.1093/gbe/evy206 (PMC6185446; doi:10.1093/gbe/evy206)
Supplement: Supplementary Data [file evy206_supp.pdf]

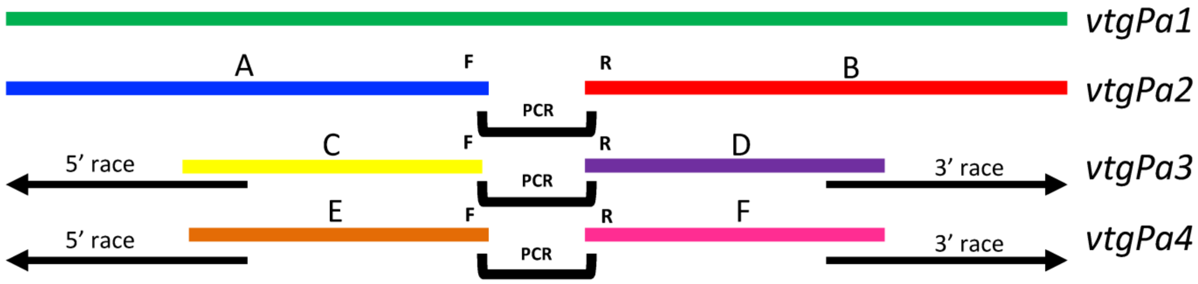


**Supplementary figure 1. Schematic representation of *vitellogenin* transcripts derived from *P. annectens* transcriptome.**

Schematic representation of vitellogenin transcripts retrieved from the *P. annectens* transcriptome and strategy applied for the reconstruction of *vitellogenin* sequences. Colored rectangles indicate complete and partial *vtg* transcripts; F and R letters located at the extremities of partial transcripts represent the primers used in PCR to cover the missing central region.

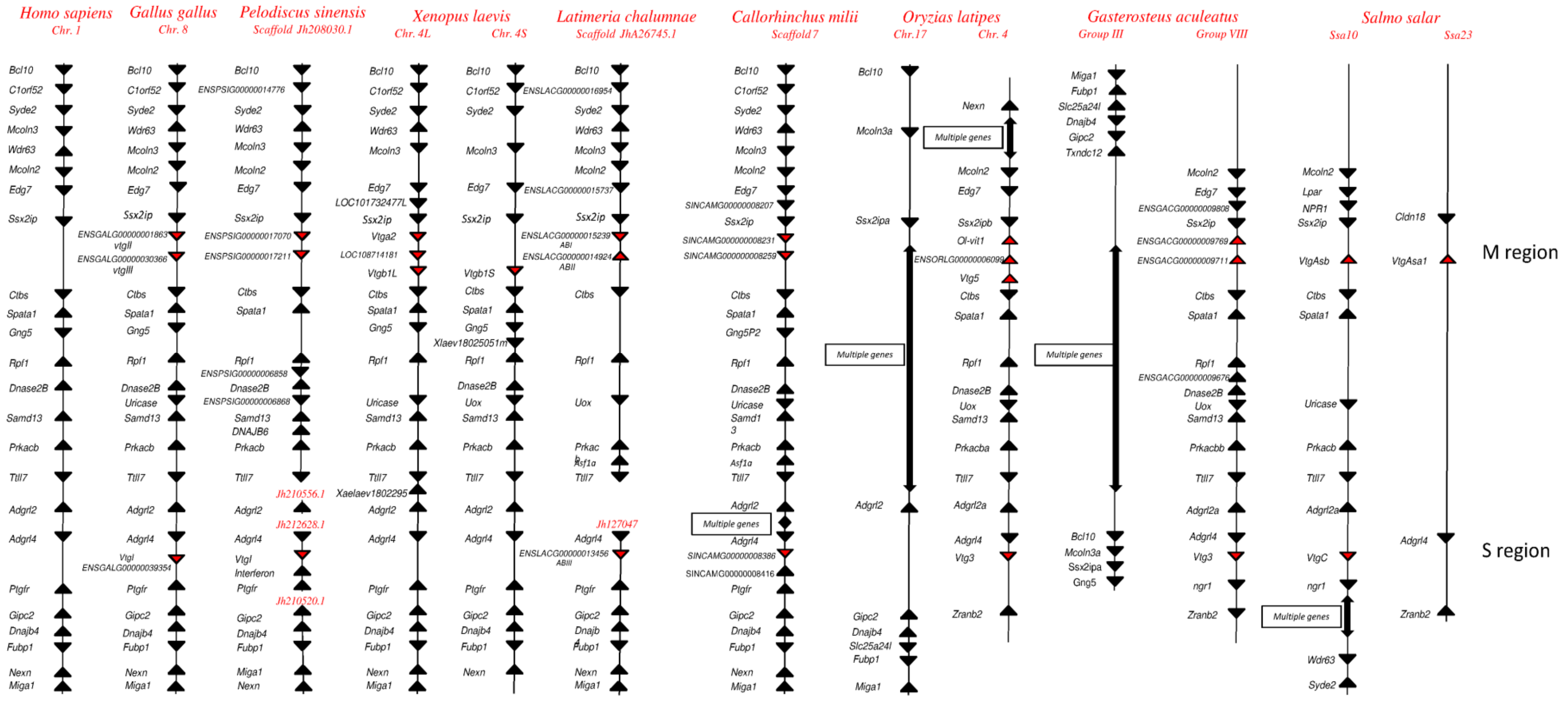


**Supplementary figure 2. Synteny conservation of *vtg* genes.**

Lines underneath genes indicate syntenic arrangement and arrowheads indicate gene direction. Syntenic maps are reported for *H. sapiens*, *G. gallus*, *P. sinensis*, *X. laevis*, *L. chalumnae*, *C. milii*, *O. latipes*, *G. aculeatus*, and *S. salar*. The gene distances are not in scale.

***Gallus gallus***

*a) Comparison between paralog genes of M locus*

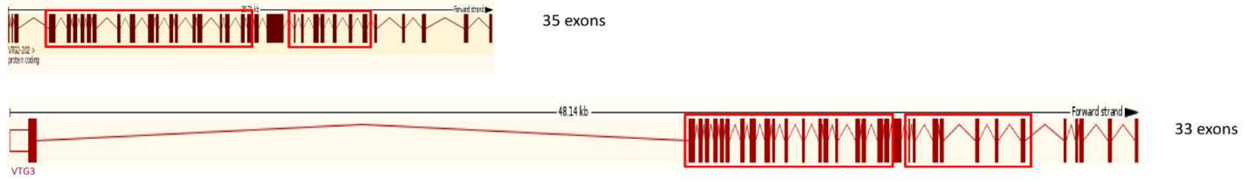


*b) Comparison between the gene of S locus with vtg2 of M locus*

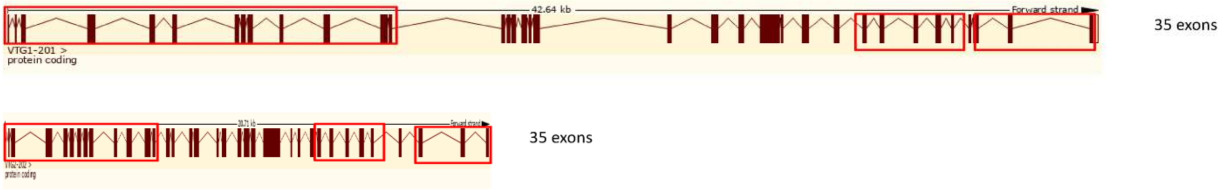


*c) Comparison between the gene of S locus with vtg3 of M locus*

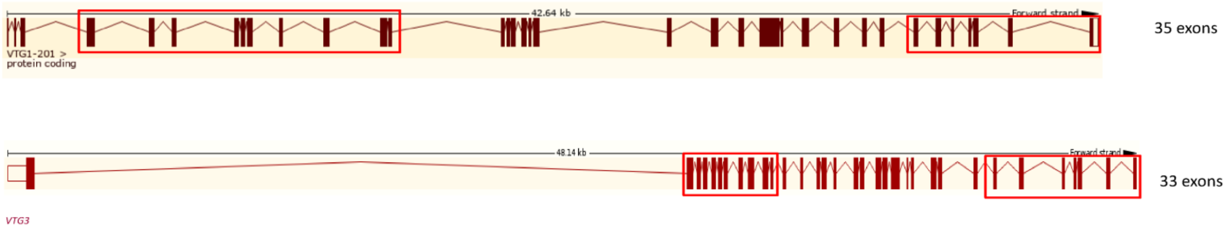


***Pelodiscus sinensis***

*a) Comparison between paralog genes of M locus*

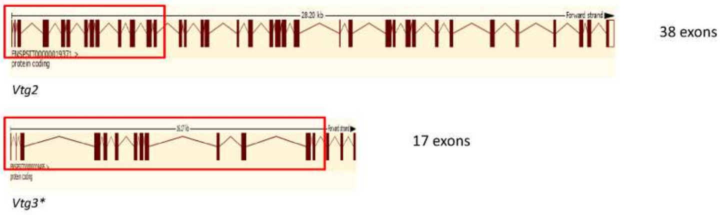


*b) Comparison between the gene of S locus with vtg2 of M locus*

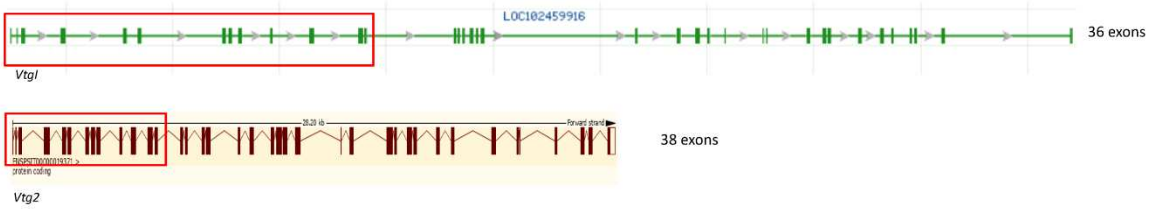


*c) Comparison between the gene of S locus with vtg3 of M locus*

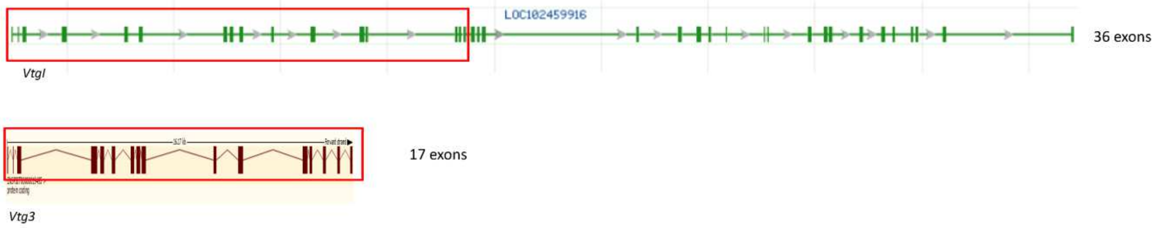

***Latimeria chalumnae***

*a) Comparison between paralog genes of M locus*

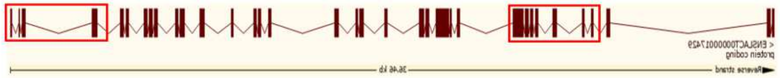

37 exons

*ABI*

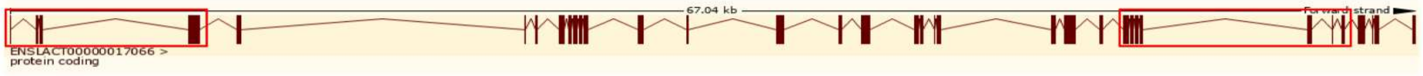


40 exons

*ABII*

*b) Comparison between the gene of S locus with vtgABI of M locus*

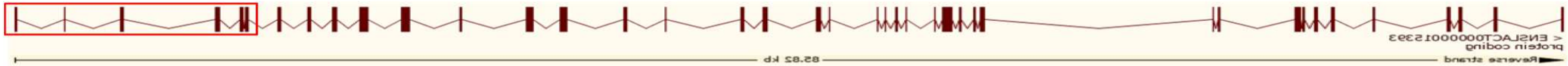

45 exons

*ABIII*

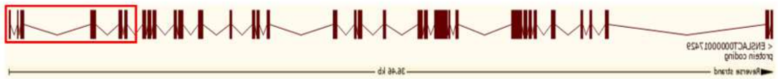

37 exons

*ABI*

*c) Comparison between the gene of S locus with vtgABIII of M locus*

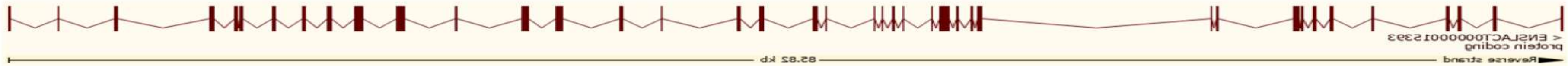

45 exons

*ABIII*

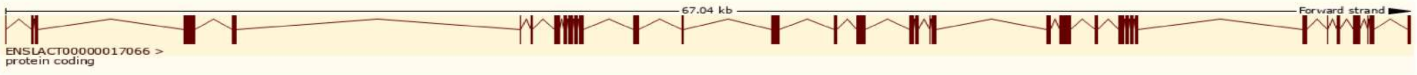


40 exons

*ABII*

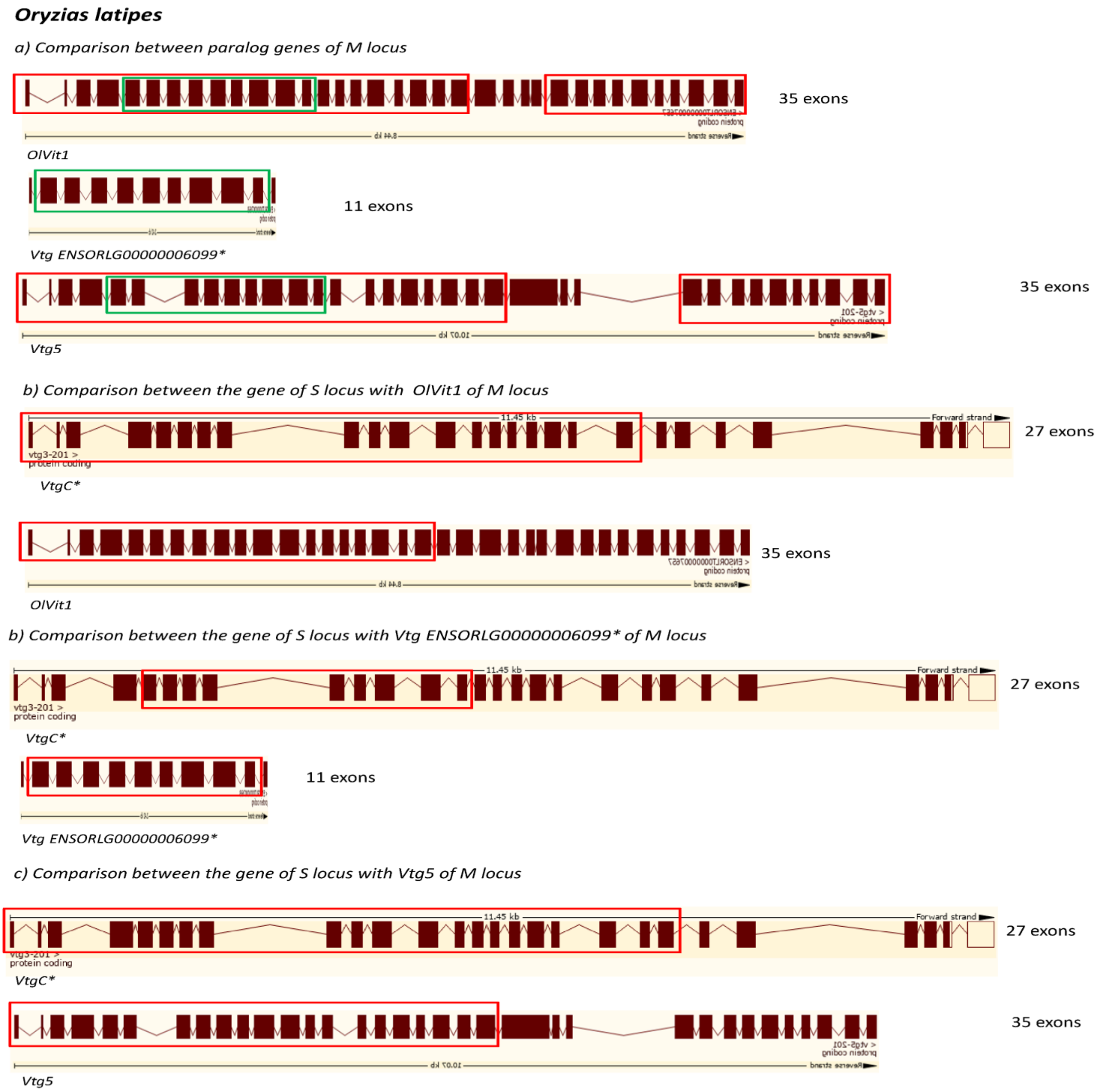


**Supplementary figure 3. Comparison of exon-intron schematic representations between *vtg* paralog genes.**

Comparison between paralog genes of M locus and between the gene of S locus with those of M locus in *G. gallus*, *P. sinensis*, *L. chalumnae*, and *O. latipes*. The red and green boxes evidence similar exon-intron patterns. * indicates incomplete sequence.

TABLE S1. List of primers used in PCR and in RACE techniques.

| Primers | Sequences |
|---|---|
| vtg A-F | 5’ CATCTAGTACAGAATAATGTGCG 3’ |
| vtg B-R | 5’ AATGGGATGGCAGCCTGAGC 3’ |
| vtg C-F | 5’ CCTCAGAAGTGCAATTGAACTGAA 3’ |
| vtg D-R | 5’ AAAGCTCATGAGAAGGATTG 3’ |
| vtg E-F | 5’AAGACAACAGCAGCAGGATCG 3’ |
| vtg F-R | 5’ACAGCTCATGAGAAAGGTTA 3’ |
| 3RACEvtgPa4_F | 5’ CCTTCAGGCTCTTTGGCACT 3’ |
| 3RACE | 5' GACTCGAGTCGACATCGATTTTTTTTTTTTTTTTTT 3' |
| 3RACEvtgPa4_F1 | 5’ AGTCCTGCTCTGAAGCTTGT 3’ |
| 3RACEvtgPa3_F2 | 5’ AGTCGTGCTCTGAAGCATGC 3’ |
| 3Raceb | 5’ GACTCGAGTCGACATCG 3’ |
| 5RACEvtgPa3-R1 | 5’ TCTGAACCACTATGTCTT G 3’ |
| 5RACEvtgPa3-R2 | 5’ TCTTGTGATCCTTCTTAGCAATG 3’ |
| 5RACEvtgPa3-R3 | 5’ TCACAGCTGCTACCTTAGTCCTG 3’ |

TABLES2. Accession numbers.

| Classe | Ordine | Specie | Vtg | Accession number |
|---|---|---|---|---|
| Mammalia | Monotremata | *Ornithorhynchus anatinus* | | XP_001513824 |
| Sauropsida | Testudines | *Pelodiscus sinensis* | 1 | XP_006136373 |
| | | | 2 | ENSPSIP00000019281 |
| | | | 3 | XP_014427569 |
| Aves | Galliformes | *Gallus gallus* | I | ENSGALP00000044130 |
| | | | II | ENSGALP00000002888 |
| | | | III | ENSGALP00000002886 |
| | Passeriformes | *Taeniopygia guttata* | 1 | XP_002188035 |
| | | | 2 | XP_002186993 |
| | | | 3 | XP_002187307 |
| | | *Ficedula albicollis* | 1 | ENSFALT00000006221 |
| | | | 2 | ENSFALT00000006454 |
| | | | 3 | ENSFALT00000006342 |
| Amphibia | Anura | *Xenopus laevis* | Vtga2 | XB-GENE-5791437 |
| | | | Vtgb1L | XB-GENE-5749200 |
| | | | Vtgb1S | XB-Gene-17342380 |
| | | | 1 | Scaffold137809:1908899..1923184<br>XM_018258149.1 |
| | | *Xenopus tropicalis* | 1 | XP_002931743 |
| | | | 2 | XP_002931744 |
| | | | A2 | XP_004913968 |
| | Caudata | *Andrias davidianus* | | AKN59013.1 |
| Sarcopterygii | Coelacanthiformes | *Latimetia menadoensis* | ABI | CCG55373.1 |
| | | | ABII | CCG55374.1 |
| | | | ABIII | CCG55372.1 |
| | | *Latimetia chalumnae* | ABI | ENSLACP00000017302 |
| | | | ABII | ENSLACP00000016946 |
| | | | ABIII | ENSLACP00000015287 |
| | | *Protopterus annectens* | 1 | MH817464 |

| | | | | |
|---|---|---|---|---|
| | | | 2 | MH817465 |
| | | | 3 | MH817466 |
| | | | 4 | MH817467 |
| Actinopterygii | Lepisosteiformes | *Lepisosteus oculatus* | Vtg5 | ENSLOCP00000012416 |
| | | | AB1 | ENSLOCP00000012351 |
| | | | C | ENSLOCP00000010349 |
| Acanthopterygii | Beloniformes | *Oryzias latipes* | Aa1 | BAB79696 |
| | | | Vtg5 | ENSORLP00000007793 |
| | | | vtg3 | ENSORLP00000008173 |
| | | | Aa2 | XP_004067780.1 |
| | Tetraodontiformes | *Takigufu rubripes* | C | ENSTRUP00000009572 |
| | | | 1 | ENSTRUP00000044803 |
| | | | 2 | ENSTRUP00000044806 |
| | Gasterosteiformes | *Gasterosteus aculeatus* | Aa | ENSGACP00000012923 |
| | | | Ab | ENSGACP00000012842 |
| | | | Vtg3 | ENSGACP00000012536 |
| | Pleuronectiformes | *Verasper moseri* | Aa | BAD93695 |
| | | | Ab | BAD93696 |
| Elopomorpha | Anguilliformes | *Anguilla japonica* | Ae1 | AAV48826 |
| | | | Ae2 | AAR82899 |
| | | | Ae3 | AAR82898 |
| Ostariophysi | Cypriniformes | *Danio rerio* | Vtg1 (Ao1) | AAK94945 |
| | | | Vtg3 (C) | AAG30407 |
| | | | Vtg4 (Ao1) | ENSDARP00000072764 |
| | | | Vtg5 (Ao1) | ENSDARP00000061222 |
| | | | Vtg6 (Ao1) | ENSDARP00000095999 |
| | | | Vtg7 (Ao2) | ENSDARP00000072678 |
| | | | Vtg8 (Ao2) | ENSDARP00000072780 |
| | | | Vtg2 (Ao2) | ENSDARP00000061164 |
| Actinopteri | Salmoniformes | *Salmo salar* | VtgAsa1 | NC_027322: 3409007-3419329 |

| | | | | |
|---|---|---|---|---|
| | | | VtgAsb | NC_027309: 1467714-1495034 |
| | | | VtgC | NC_027309: 3287395-3335002 |
| Chondrostei | Acipenseriformes | *Acipenser schrenckii* | AB1 | AOH96642.1 |
| | | | AB2a | AOH96643.1 |
| | | | AB2b | AOH96644.1 |
| Hyperoartia | Petromyzontiformes | *Ichthyomyzon unicuspis* | | AAA49327 |
| | | *Petromyzon marinus* | | GL478968:39107-09216 |
| Chondrichthyes | Chimaeriformes | *Callorhinchus milii* | 1 | ENSCAMG00000008231 |
| | | | 2 | ENSCAMG00000008259 |
| | | | VtgC | ENSCAMG00000008386 |
